# ‘Transparent mice’: deep-tissue live imaging using food dyes

**DOI:** 10.1038/s42003-024-07012-9

**Published:** 2024-10-11

**Authors:** Fei Wang, Chao Zhou

**Affiliations:** Department of Biomedical Engineering, Washington University in St. Louis, Saint Louis, MO USA

## Abstract

Light scattering, resulting from refractive index mismatch, is the primary factor limiting imaging depth in biological tissue. In a study recently published in *Science*^1^, Ou et al. took an unconventional approach and employed highly absorbing molecules, such as food dye tartrazine, to increase the refractive index of water in the near-infrared wavelength range, which rendered tissue transparent in live mice.

Light-based techniques have transformed modern biology and medicine, enabling advances in sensing, diagnostics, imaging, and light-activated therapies^2^. However, light scattering poses significant challenges in biological applications, particularly for deep-tissue optical imaging. Biological tissues comprise various cellular structures within an aqueous matrix, such as organelles, proteins, and lipids, each with different refractive indices (RIs). Their inhomogeneous nature causes photons to deviate from straight paths, reducing light intensity and image resolution at greater depths^3^. Tissue-clearing techniques aim to match RIs, mostly by removing high-RI scatterers like lipids from the low-RI water environment^4^. However, only a few agents, such as glycerol, are suitable for in vivo use due to the need for biocompatibility.

Now, Ou and colleagues provide an unconventional solution using strongly absorbing molecules, expanding the in vivo clearing toolbox^1^. These water-soluble dyes can significantly raise the RI of an aqueous medium, efficiently reducing the scattering events, particularly at longer wavelengths. For example, tartrazine, a yellow-to-orange synthetic food dye with high blue absorbance, is two to three orders of magnitude more efficient than other common optical clearing agents at raising water’s RI at the near-infrared (NIR) wavelengths. Current agents like glycerol are inefficient at changing the RI and require high concentrations that can cause tissue shrinkage and distortion, making them unsuitable for in vivo use. According to the Lorentz oscillator model and the Kramers-Kronig relations^1^, strongly absorbing molecules in the near-ultraviolet to blue wavelength range can increase the RI of the medium in the NIR range much effectively than most optical clearing agents. The authors conducted dye screening and discovered additional dyes achieving better transparency than tartrazine in the NIR spectrum.

Furthermore, the authors explored the use of tartrazine to make mouse skin, muscle, and connective tissues transparent^1^. A transparent abdomen was demonstrated by applying tartrazine, enabling direct visualization of gut movements in vivo. Tartrazine was also applied to the scalp for visualization of cerebral blood vessels using laser speckle imaging, and to the hindlimb for microscopic imaging of muscle sarcomeres. Tissue transparency can be reversed by rinsing off the dyes after experiments.

To achieve deep-tissue imaging, researchers have explored various innovative approaches. Multiphoton microscopy relies on the non-linear absorption of NIR light, which experiences lower tissue scattering, allowing for deeper imaging depth^5^. Optical coherence tomography detects ballistic photons, rather than multiply-scattered light, achieving deep tissue imaging through high-detection sensitivity^6^. Photoacoustic tomography combines the benefits of specific absorption of light and weak-scattering of ultrasound to localize deep signals^7^. Diffuse optical spectroscopy and tomography utilize a diffusion model to describe light propagation in highly scattering tissue, allowing for the probing of deep tissue physiology at a cost of reduced resolution^8^. Finally, wavefront shaping methods engineer the light wave’s wavefront to achieve optical focusing deep within scattering tissue^9^. As a complementary approach to these existing methods, absorbing dyes can be used in conjunction with these techniques to further improve imaging depth and quality in live tissue in a non-invasive manner.

This in vivo tissue clearing approach holds great potentials for advancing a wide range of light-based techniques. Beyond imaging, it can improve light-based treatments like photodynamic therapy^10^, as well as optical stimulation technologies such as optogenetics^11^. Advancements in developing new biocompatible dyes with high clearing efficiency across a broad wavelength range, along with strategies to enhance dye diffusion, will further expand the adoption of this technology. As we begin to peer through an intact and transparent window enabled by highly absorbing dyes in live animals, new biological insights are expected to emerge.
